# Prevalence and pattern of vitreo-retinal diseases in Nepal: the Bhaktapur glaucoma study

**DOI:** 10.1186/1471-2415-13-9

**Published:** 2013-03-28

**Authors:** Suman S Thapa, Raba Thapa, Indira Paudyal, Shankar Khanal, Jaskirat Aujla, Govinda Paudyal, Ger van Rens

**Affiliations:** 1 Nepal Glaucoma Eye Clinic, Tilganga Institute of Ophthalmology, Kathmandu, Nepal

**Keywords:** Vitreo-retinal disorders, Prevalence, Low vision, Blindness, Nepal

## Abstract

**Background:**

Vitreo-retinal diseases are among the leading causes of visual impairment and blindness worldwide. This study reports the prevalence and pattern of vitreo-retinal diseases in the Bhaktapur Glaucoma Study (BGS), a population based study conducted in Nepal.

**Methods:**

BGS was a population based cross-sectional study involving 4800 subjects aged 40 years and over from Bhaktapur district. Subjects were selected using a cluster sampling methodology and a door-to-door enumeration. All subjects underwent a detailed ocular examination at the base hospital which included log MAR visual acuity, refraction, applanation tonometry and a dilated fundus examination. Fundus photography, optical coherence tomography and fundus fluorescein angiography were performed where indicated.

**Results:**

Complete data was available for 3966 (82.62%) out of the total of 4800 enumerated subjects. The mean age was 55.08 years (SD 11.51). The overall prevalence of vitreo-retinal disorders was 5.35% (95% CI, 4.67 - 6.09). Increasing age was associated with a higher prevalence of vitreo-retinal disorders (P < 0.001). The prevalence of diabetes mellitus was 7.69% (95% CI, 6.88 - 8.56). Age-related macular degeneration (AMD) was the most common vitreo-retinal disorder with a prevalence of 1.50% (95% CI, 1.15 - 1.94), increasing significantly with age. The prevalence of diabetic retinopathy among the study population was 0.78% (95% CI, 0.53 - 1.11) and among the diabetic population 10.16% (95% CI, 7.01 - 14.12). The population prevalence of other retinal disorders were hypertensive retinopathy 0.88%, macular scar 0.37%, retinal vein occlusion 0.50%, macular hole 0.20%, retinitis pigmentosa 0.12%. and retinal detachment 0.10%.

The prevalence of low vision and blindness due to vitreo-retinal disorders was 1.53% (95% CI, 1.18 - 1.97) and 0.65% (95% CI, 0.43 - 0.96), respectively. The prevalence of low vision and blindness was 28.77% (95% CI, 22.78-35.37) and 12.26% (95% CI, 8.17-17.45), respectively among cases with vitreo-retinal disorders. Blindness was observed to be unilateral in 19 cases (73%), and bilateral in 7 cases (27%).

**Conclusions:**

The prevalence of vitreo-retinal disorders in this Nepalese population was 5.35%, which increased significantly with age. AMD was the predominant retinal condition followed by diabetic retinopathy. One fourth of the subjects with vitreo-retinal disorder had low vision. Taking into consideration the aging population and emerging systemic diseases such as diabetes mellitus and hypertension, vitreo-retinal disorders could be of future public health importance.

## Background

Vitreo-retinal diseases as a group are one of the more common ocular morbidities leading to blindness in the adult population, while being the most common cause of blindness worldwide in children [1]. Population based studies reported an overall prevalence of vitreo-retinal disorders of 8.56%, with a range between 10.4% and 21.02% for the 40 years and over age group [2,3]. Age related macular degeneration (AMD) is the third commonest cause of blindness globally and is the leading cause of irreversible blindness among the elderly in developed countries, contributing to 8.7% of total blindness [4-7]. Likewise, diabetic retinopathy (DR) is the fifth leading cause of visual impairment and blindness globally, and is the most common cause of new cases of blindness among working aged adults in the developed world [4,8-10]. In the developing world, which harbours almost 90% of the world’s blind population, retinal diseases are among the leading cause of blindness after cataract [11]. In Asia, vitreo-retinal diseases are becoming an increasing problem, with expectations that more than half of the world’s diabetic patients will live in Asian countries by the year 2030 [12-17].

The Nepal Blindness Survey of 1981 reported retinal disorders to be the third leading cause of bilateral blindness after cataract and its sequelae [18]. After the 1981 survey there have been several population based studies in Nepal that have reported the prevalence of blindness from retinal diseases within a range of 1% to 10.8% [19,20]. Based on these studies, it has been reported that AMD and retinal detachment are the major causes of visual impairment.

A recent population based study conducted in the Bhaktapur district reported that vitreo-retinal diseases were the second major cause of blindness following cataract, while being the most common cause of blindness among pseudophakics [21,22]. This paper will further elaborate the findings from this population based study regarding the prevalence, types and visual impairment from vitreo-retinal disorders in this district. While previous studies have only reported on the overall prevalence of blindness from eye disorders including retinal diseases, this will be the first study to report the prevalence and pattern of vitreo-retinal disease in Nepal.

## Methods

The methods and demographics of the study population have been published in detail elsewhere [23]. In brief; the survey involved the selection of 4800 subjects aged 40 years or older residing in the Bhaktapur district. Bhaktapur is situated approximately 15 kilometres away from the capital city of Kathmandu. It is located within the mid-mountain region of Nepal. A sample size of 4758 was calculated after assuming a prevalence of 3 % for glaucoma based on studies in the region, a relative precision of 25%, 85% compliance and a design effect of 2. A total of 4800 subjects were enumerated. A 2-stage World Health Organization 30-cluster sampling procedure was adopted for patient selection [24]. The sampling frame comprised 161 wards and an estimated population of 48,223 people aged over 40 years [25]. In the first stage, 30 wards were selected and field workers conducted a census. In the second stage, a database was prepared and names of all eligible subjects were recorded, and then subjects were selected randomly. From this list, 4800 subjects were selected using EPI-INFO software, version 3.5.1 (Centers for Disease Control and Prevention, Atlanta, GA). Selected subjects were revisited by field workers and referred to Tilganga Institute of Ophthalmology for a comprehensive eye examination. The study was approved by the institutional review board of Tilganga Institute of Ophthalmology and conducted in accordance with the Declaration of Helsinki. Informed consent was obtained from all subjects after explaining all the procedures to be undertaken. The informed consent was written in the vernacular. For those unable to read, the consent form was read out to them. Upon agreement by the subject, they were asked to sign the consent form. For those unable to sign, thumb impressions were taken. Bhaktapur district was chosen because it did not have an eye hospital and for logistic reasons in order to conduct a large population based study.

### Clinical examination

Two ophthalmologists were involved in the clinical examination of all the subjects as per the protocol [23]. Patients with vitreo-retinal disorders were referred to a fellowship trained vitreo-retina specialist for confirmation of diagnosis and further management. All referred patients were examined by the vitreo -retinal (VR) specialist on the same day. This was possible as the study was conducted patients at a tertiary eye hospital where the glaucoma and retina units work on the same days of the week.

The visual acuity (VA) was measured using logarithm of minimum angle of resolution (logMAR) with tumbling E charts placed at 4 metres. Slit-lamp biomicroscopy (BQ 900, Haag-streit International, Koeniz, Switzerland) was performed to identify ocular abnormalities. Fundus evaluation was performed with the aid of 90 D and 20 D lens (Volk optical Inc, Mentor, OH0). All subjects had their pupils dilated. Those with risk of angle closure underwent peripheral iridectomy before pupil dilatation. The lens was examined and cataract graded according to lens opacities Classification System 2 (LOCS II) [26]. Fundus examination included evaluation of the optic disc, macula, vessels, and central and peripheral retina.

AMD was graded according to the International classification developed by the International ARM Epidemiological Study Group [27]. Briefly, drusen were defined as discrete whitish-yellow spots external to the neuroretinal pigment epithelium or retinal pigment epithelium (RPE). The largest drusen determined the grade for maximum drusen size and predominant drusen type. Type was based on the size of drusen, uniformity of appearance across the breadth, and the sharpness of edges. Pigmentary abnormalities included either increased pigmentation associated with drusen or depigmentation or hypopigmentation of the RPE, more sharply demarcated than drusen, without any visibility of choroidal vessels associated with drusen. Geographic atrophy was defined as any sharply delineated, approximately round or oval area of hypopigmentation or depigmentation, or apparent absence of the RPE in which choroidal vessels are more visible than in surrounding areas, at least 175 μm. Wet AMD was defined as the presence of any of the following: (1) RPE detachments or serous detachment of the sensory retina, (2) sub retinal or sub-RPE neovascular membranes, (3) subretinal haemorrhages and (4) epiretinal, subretinal, intraretinal, or sub pigment epithelial scar or glial tissue or fibrin like deposits.

The assessment of diabetes mellitus was based on either the use of diabetic medications or a random blood sugar level of 200 mg/dl or greater. Glycosylated haemoglobin was not measured.

DR was categorized as per the Early Treatment Diabetic Retinopathy Study [28]. Briefly, DR was classified into various stages of non proliferative (NPDR) and proliferative DR. Clinically significant macular oedema was defined as retinal oedema located at or within 500 μm of the centre of the macula or hard exudates at or within 500 μm of the centre, if associated with thickening of adjacent retina, or a zone of thickening larger than 1 disc area if located within 1 disc diameter of the centre of the macula.

We defined systemic hypertension as either a measured systolic blood pressure of 140 mmHg or greater, or a diastolic blood pressure of 90 mmHg or greater, or current use of systemic antihypertensive medications.

Fluorescein angiography, Optical Coherence Tomography (OCT) and surgical interventions were carried out based upon the expert opinion of the VR surgeon. FFA and OCT were also done as a baseline to evaluate the treatment outcome.

The *International Classification of Diseases* 10^th^ edition defines visual impairment as VA less than 6/18 (20/60, 0.3) in the better eye with best correction [29]. Visual impairment has been categorized into blindness and low vision. A VA of less than 3/60 (20/400, 0.05) with best correction in the better eye has been considered blindness. Low vision has been defined as a best-corrected VA of less than 6/18 (20/60, 0.3) but not less than 3/60 (20/400, 0.05) in the better eye. VA was based on the eye with vitreo-retinal disease in unilateral cases and on the better eye in bilateral cases. The prevalence of disease was based upon the number of subjects and not eyes. So if subjects had glaucoma in one eye and a retinal pathology in the other, they were counted separately.

### Statistical analysis

Descriptive measures such as mean, standard deviation for continuous variables, ratios and percentages for categorical variables were computed. The prevalence estimates with 95% confidence intervals were calculated using binomial distribution. Univariate and multivariate logistic regression analysis was carried out to see the association between the probable risk factors with vitreo-retinal diseases. Statistical analysis was performed using STATA 9.0 (Stata Corporation, College Station, Texas, U.S.A.).

## Results

Complete data was available for 3966 (82.62%) out of the total of 4800 subjects. The mean age was 55.08 years (SD 11.51), with a range from 40 years to 96 years. Males comprised of 45.51% and females 54.49% of the total study group. The total number of non-responders was 834 (46% males and 54% females). The mean age of non-responders was 54.72 years (SD 10.92) which was not statistically significant in comparison to the responders.The overall prevalence of various vitreo-retinal disorders was 5.35% (95% CI, 4.67 - 6.09). Increasing age was associated with a higher prevalence of vitreo-retinal disorders (P < 0.001), as shown in Table 1. The prevalence was 1.96% (95% CI, 1.32 – 2.80) for the age group 40–49 years, compared to 28.57% (95% CI, 3.67 - 70.90) for the age group 90 years and above. There was no significant difference in the age-adjusted prevalence of vitreo-retinal disorders between sexes.

**Table 1:** Age and sex distribution of vitreo-retinal diseases in the study population

**Characteristics**	**No. of participants**	**Prevalence% V-R disorders (95% CI)**	**Crude OR (95% CI)**	**Adjusted OR (95% CI)**
Age				
40 - 49	1477	1.96 (1.32, 2.80)	1.00	1.00
50 - 59	1080	4.54 (3.37, 5.95)	2.37 (1.49, 3.78)	2.38 (1.49, 3.79)
60 - 69	837	7.05 (5.41, 8.99)	3.79 (2.40, 5.96)	3.81 (2.42, 5.99)
70 - 79	475	12.42 (9.59, 15.73)	7.08 (4.48, 11.19)	7.10 (4.49, 11.22)
80 - 89	90	15.60 (8.77, 24.72)	9.19 (4.67, 18.12)	9.20 (4.67, 18.14)
≥90	7	28.57 (3.67, 70.9)	19.97 (3.72, 107.22)	20.24 (3.76, 108.78)
Sex				
Male	1805	5.20 (4.23, 6.34 )	1.00	1.00
Female	2161	5.46 (4.54, 6.50)	1.05 (0.79, 1.39)	1.09 (0.82, 1.45)
Total	3966	5.35 (4.67, 6.09)	-	-

No, number; CI, confidence interval; OR, odds ratio; V-R, vitreo-retinal.

Table 2 shows the pattern of various vitreo-retinal disorders seen in this population. AMD (28.3%) was the most common vitreo-retinal disorder. The prevalence of AMD was 1.5% (95% CI, 1.15-1.94); 1.38% (95% CI, 0.89-2.04) was noted in males and 1.62% (95% CI, 1.13-2.24) in females. The mean age of subjects with AMD was 69.28 years (SD 12.31). Table 3 shows the prevalence of AMD in different age groups. There was a significant association between AMD and increasing age. Sex was not a risk factor for AMD.

**Table 2:** Pattern of vitreo-retinal disorders in Bhaktapur

**Vitreo-retinal diseases**	**Disease pattern**	**Persons. No. (%)**	**Eyes. No. (%)**
Macular lesions	ARMD	60 (28.30)	113 (31.56)
	Macular scar	15 (7.07)	17 (4.74)
	Macular hole	8 (3.77)	10 (2.79)
	ERM	2 (0.94)	2 (0.55)
	Macular dystrophy	1 (0.47)	2 (0.55)
Retinal lesions	Diabetic retinopathy	38 (17.90)	76 (21.20)
	HTN Retinopathy	35 (16.50)	70 (19.50)
	BRVO	19 (8.96)	19 (5.30)
	Retinitis pigmentosa	5 (2.36)	10 (2.79)
	Retinal detachment	4 (1.89)	4 (1.10)
	Chorio-retinal scars	4 (1.89)	4 (1.10)
	Myopic fundus	2 (0.94)	2 (0.55)
	Central retinal vein occlusion	1 (0.47)	1 (0.27)
	Coloboma	1 (0.47)	1 (0.27)
	Macular dystrophy	1 (0.47)	2 (0.55)
Vitreous	Vitreous hemorrhage	1 (0.47)	1 (0.27)
	PVD	9 (4.24)	11 (3.07)
	Asteroid hyalosis	1 (0.47)	8 (2.23)
Others		5 (2.35)	5 (1.39)
Total		212	358

No: number, ARMD: age-related macular degeneration, ERM: epiretinal membrane, HTN: hypertension, BRVO: branch retinal vein occlusion, PVD: posterior vitreous detachment.

**Table 3:** Prevalence of age-related macular degeneration, diabetes and diabetic retinopathy

**Age group**	**Prevalence(% ) with CI of AMD among study population**	**Prevalence(% ) with CI of diabetes mellitus among study population**	**Prevalence(% ) with CI of diabetic retinopathy among study population**	**Prevalence(% ) with CI of diabetic retinopathy among diabetic subjects**
40-49	0.27 (0.073-0.69)	4.74 (3.71-5.95)	0.61 (0.28 - 1.15)	12.86 (6.05 - 23.00)
50-59	0.56 (0.20-1.20)	7.96 (6.42-9.74)	0.83 (0.38 - 1.58)	10.46 (4.89 - 18.94)
60-69	1.90 (1.09-3.08)	10.63 ( 8.63-12.92)	1.79 (1.00 - 2.94)	16.85 (9.75 - 26.27)
70-79	5.05 (3.26-7.42)	10.74 (8.09-13.87)	0.84 (0.23 - 2.14)	7.84 (2.18 - 18.88)
80-89	8.89 (3.92-16.76)	7.78 (3.58-15.37)	1.11 (0.03 - 6.04)	14.28 (0.36 - 57.87)
≥90	28.57 (3.67-70.96)	28.57 (3.67-70.95)	No cases	

(Diabetic retinopathy n= 38; total diabetic subjects n= 305).

DR comprised of 17.9% of the vitreo-retinal disorders, which was second to AMD. The prevalence of diabetes mellitus was 7.69% (95% CI, 6.88 - 8.56) in the study population, out of which known and newly diagnosed diabetics comprised of 6% (95% CI, 5.28 - 6.79) and 1.69% (95% CI, 1.31 - 2.14), respectively. The overall prevalence of DR was 0.78% (95% CI, 0.53 - 1.11) among the study population and 10.16% (95% CI, 7.01 - 14.12) among the diabetic population. The prevalence of diabetes and DR among the general and diabetic population is shown in Table 3. The prevalence of diabetes was higher with increasing age. The prevalence of NPDR was 0.93% (95% CI, 0.65 - 1.28) in our study population. There were only two cases with clinically significant macular oedema and one case with proliferative DR (Table 2,3).

The other common vitreo-retinal disorders were hypertensive retinopathy (16.5%), branch retinal vein occlusion (8.96%), macular scar (7.07%) and retinal detachment (1.89%) among the vitreo-retinal disorders.

The prevalence of various grades of hypertensive retinopathy was 0.88%, retinal vein occlusion 0.5%, and branch retinal vein occlusion 0.47%. There was only one case with central retinal vein occlusion. The prevalence of other sight threatening vitreo-retinal diseases was macular scar 0.37%, macular hole 0.2%, retinitis pigmentosa 0.12% and retinal detachment 0.1%. Posterior vitreous detachment was found in 0.22% of cases (Table 2).

Table 4 shows the VA distribution among the subjects with vitreo-retinal disorders. Low vision was found in 28.8% (95% CI, 22.78 - 35.37), while 12.26% (95% CI, 8.17 - 17.45) of cases were blind. The blindness was unilateral in 19 cases (73%) and bilateral in 7 cases (27%). Prevalence of low vision and blindness due to vitreo-retinal disorders was 1.54% (95% CI, 1.18 - 1.97) and 0.65% (95% CI, 0.43 - 0.96) respectively. Both low vision and blindness were more prevalent in the older age groups. The prevalence of blindness was higher among females than males. Among those with AMD, 40% had low vision and 8.33% were blind. Low vision and blindness among the cases with DR was 10.5% and 2.6% respectively. Likewise, 42.10% of cases had low vision and 15.78% were blind among the cases with branch retinal vein occlusion. The rate of blindness was high (75%) among cases of retinal detachment.

**Table 4:** Visual acuity distribution among cases with vitreo-retinal disorders

**Characteristics**	**Number of cases with vitreo-retinal diseases**	**Normal vision No. (%)**	**Low vision No. (%)**	**Blind No. (%)**
**Age (years)**				
40 - 49	29	24 (82.75)	4 (13.79)	1(3.40)
50 - 59	49	31 (63.26)	9 (18.36)	9 (18.36)
60 - 69	59	39 (66.10)	16 (27.10)	4 (6.80)
70 - 79	59	27 (45.76)	25 (42.37)	7 (11.86)
80 - 89	14	4 (28.57)	5 (35.71)	5 (35.71)
≥90	2	0 (0.00)	2 (100.00)	0 (0.00)
**Sex**				
Male	94	62 (65.90)	23 (24.40)	9 (9.50)
Female	118	63 (53.38)	38 (32.20)	17 (14.40)
**Vitreo-retinal diseases**				
AMD	60	31 (51.67)	24 (40%)	5 (8.33)
Diabetic retinopathy	38	33 (86.80)	4 (10.50)	1 (2.60)
Hypertensive retinopathy	35	30 (85.70)	5 (14.30)	0
Branch retinal vein occlusion	19	8 (42.10)	8 (42.10)	3 (15.78)
Macular scar	15	5 (33.33)	5 (33.33)	5 (33.33)
Macular hole	8	3 (37.50)	4 (50)	1 (12.5)
Retinitis pigmentosa	5	0	4 (80)	1 (20)
Retinal detachment	4	0	1 (25)	3 (75)
Others	28	15 (53.57)	6 (21.40)	7 (25)
**Total**	212	125 (59)	61 (28.80)	26 (12.20)

**Note:** Visual acuity was based on the eye with vitreo-retinal diseases in unilateral cases and on the better eye in bilateral cases. Low vision has been defined as a best-corrected visual acuity of <6/18 (20/60, 0.3) but not <3/60 (20/400, 0.05).

## Discussion

This is the first population based study to report the prevalence of vitreo-retinal disorders in Nepal. Our findings are not representative of Nepal because of the diverse ethnic races seen in the country that could play a role in the etiology of various eye diseases. However considering the similarity in socioeconomic conditions, geographic terrain and the ethnic races with the neighbouring two districts, our findings could be representative of Kathmandu valley. The findings of this study will be useful for gauging the pattern of vitreo-retinal disorders in the Bhaktapur district, and thus assist with guiding future public health interventions for the area. It also importantly has the potential to be extrapolated to the two neighbouring districts of Kathmandu and Lalitpur, due to similar demographics and geography. The overall prevalence of vitreo-retinal disorders in this study was 5.35%, a figure which is lower than some of the reports from around the region [2,3]. There lies the possibility in our study for an underestimation of the overall prevalence of vitreo-retinal disorders, due to the fact the original design was primarily for the study of glaucoma. In addition, the inability to perform fundus photography on all subjects may have also lead to an underestimation. Despite these factors, the prevalence of vitreo- retinal disorders was higher than the overall prevalence of glaucoma (1.2%). In investigating the prevalence of vitreo-retinal disorders and its association with age and gender, we found there to be a significant increase in prevalence with age (P<0.001), while there was no significant difference with gender in our study. These findings are consistent with those previously reported in the literature [2,3].

AMD has been the most common vitreo-retinal disease found in previous studies [2,3]. As in developed nations of the world, the increased prevalence of AMD in developing countries such as Nepal is likely to be due to an ever increasing ageing population and improving health facilities [30]. Other factors potentially related could be exposure to smoking, as well as unprotected exposure to ultraviolet radiation. This latter is a pertinent issue in Nepal where the majority of the population work in fields without adequate protective eye wears [31-33]. The prevalence of AMD in our study (1.5%) was similar to the findings of a previous study (1.8%) conducted among those over 40 years in the Indian State of Andhra Pradesh [34], but lower than other studies such as the Aravind comprehensive eye study (2.5% in the over 40 years age group) and the India Eye study (3.8% in the over 50 years age group) [3,35]. A study from China by Wu et al. showed a prevalence of 10.6% among those over 50 years [36]. Similarly studies from other developed countries have showed the prevalence of AMD to range from 8-20% [37-39]. The higher prevalence in those countries could be due to racial variations, as well as the longevity of life of people in those regions. The prevalence of AMD in our study was higher with increasing age, a finding consistent with the above mentioned studies. These findings emphasise that with longevity, AMD could become a significant public health problem in Nepal. As with other population based studies [2,3], we did not find statistically significant differences in the prevalence of AMD with gender.

The prevalence of diabetes mellitus in our study (7.69%) was comparable to that previously published in the literature [40,41]. Demonstrating a range in published data, one previous population based study conducted in a semi-urban community of Nepal, found a lower prevalence of 4.1% [42], while other studies from the region have presented a higher prevalence [41,43]. This disparity in the prevalence of diabetes could be due to the fact that our study population is predominantly involved in agriculture, where a relatively high level of physical activity may play a role in the lower incidence of diabetes. DR (prevalence of 10.16%) among our diabetic population, as well as in our overall study population (0.78%) was higher than those found in India, the study of Nirmalan et al. [3], but lower than other population based studies ranging from 18% to 63% [10,40-43]. Reasons for our lower prevalence could include a better glycemic control in this population due to better access to health care services. The higher prevalence of diabetes with increasing age was consistent with the series from India [3]. We found a higher prevalence of DR among the age group 40–49 years in our series despite a comparably lower prevalence of diabetes. This observed finding alerts to the importance of regular and timely ocular examinations in the relatively younger population.

The prevalence of hypertensive retinopathy (0.88%) in a significant proportion of our population was in keeping with the findings of previous population based studies, reflecting that hypertension is a major public health concern in Nepal [44,45]. Retinal vein occlusion on the contrary had a prevalence of 0.5% which was lower than figures from a recent Japanese study (2.1%) [46] and some other studies conducted in the western world [47,48] but similar to findings from a study in Asia [49] Macular hole, another sight threatening retinopathy, was found in our study population to have a prevalence of 0.2%, compared to a lower prevalence in the Beijing eye study (0.09%) [50], the Aravind comprehensive eye study [3], and a study by Sen et al. (0.16%) [51]. This may have been contributed by ocular trauma, especially in the younger age groups. The prevalence of retinitis pigmentosa, was not different from the study conducted by Sen et al. [52] but lower than that reported in the Aravind comprehensive eye study [3]. The high number of retinal detachments in our population indicates the need for increased awareness, education and prompt ocular assessment because of its severe visual consequences with delayed treatment. Factors that may affect rates of retinal detachment include improper management of intra-operative complications following cataract surgery and complications related to significant ocular trauma. Similarly, rates of macular scar in our population would be a result of trauma and sequelae of intraocular inflammation.

Almost two-fifths of eyes with vitreo-retinal disorders suffered from visual impairment, while blindness was present in 12.2% of eyes. The blindness was bilateral in one quarter of cases. The higher prevalence of visual impairment (1.54%) and blindness (0.65%) due to vitreo-retinal disorders among those over 40 years of age emphasises the need for routine eye examinations beginning at this age group and also promoting awareness of eye diseases. AMD, sequelae of retinal vein occlusion, retinal detachment and macular scar were the major causes of blindness in our study. The rate of visual impairment and blindness from vitreo-retinal disorders in our study was consistent with findings from previous studies conducted in Nepal [18-22]. A higher rate of blindness among females in our study could probably be due to the fact that males had better access to health care services than females.

## Conclusions

The prevalence of vitreo-retinal disorder in our population was 5.35%. AMD, DR, hypertensive retinopathy and retinal vein occlusion were the major retinal problems. The prevalence of vitreo-retinal disorders increased with age and reflects that retinal disorders will be a major public health concern with longevity in Nepal. Promoting awareness in the population in regard to regular eye examination and also addressing risk factors that could lead to vitreo retinal disorders will play a significant role in reducing blindness from this group of eye diseases.

## Competing interests

The authors declare that they have no competing interest.

## Authors’ contribution

TSS and TR: Prepared the manuscript. TR, PI, KS, AJ, PG, RGHMB: were involved in the analysis and critical review of the manuscript. All authors read and approved the final manuscript.

## Pre-publication history

The pre-publication history for this paper can be accessed here:

http://www.biomedcentral.com/1471-2415/13/9/prepub
